# Re: Digital compression of facial arteries facilitates cutaneous nasal surgery

**DOI:** 10.1111/bjd.12464

**Published:** 2013-12-02

**Authors:** S HAWORTH, G KNEPIL

**Affiliations:** Gloucestershire Hospitals NHS Foundation Trust, Gloucestershire Royal HospitalGreat Western Road, Gloucester GL1 3NN, U.K

Dear Editor, We read with interest the technique described recently by Moran *et al*.^1^ for controlling intraoperative haemorrhage during cutaneous nasal surgery.

We would like to suggest a similar technique that we have found useful in our department for skin surgery in the temporal region.

The superficial temporal artery runs through the temporoparietal fascia, and supplies a wide region of soft tissue superficial to the temporal fascia via the frontal and parietal branches. The trunk of this artery can be found reliably by palpation anterior and superior to the tragus, superficial to the root of the zygomatic arch.

Firm digital pressure at this point reduces bleeding from the proximal wound margin of excisions in the temporoparietal region. This can be achieved without discomfort to the patient (Figs 1 and 2) and facilitates haemorrhage control. A short video clip (Video S1; see Supporting Information) is included to illustrate the efficacy of this technique.

**Fig 1:**
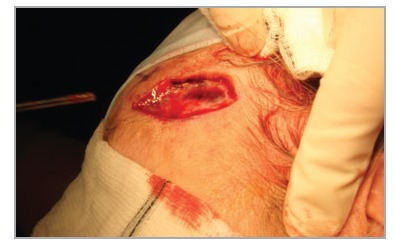
Firm digital pressure applied to the trunk of the artery facilitates haemostasis.

**Fig 2:**
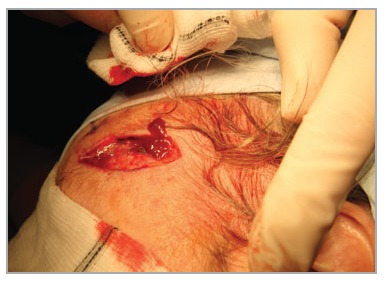
Release of pressure results in haemorrhage from the proximal wound margins.

## Supporting Information

Video S1 Digital pressure.
