# Supplementary material for: Re: Digital compression of facial arteries facilitates cutaneous nasal surgery
Source: Br J Dermatol. 2013 Dec 2;169(6):1357–60. doi: 10.1111/bjd.12464 (PMC3967071; doi:10.1111/bjd.12464)
Supplement: Related texts

# Resolution of the plantar hyperkeratosis of pachyonychia congenita during chemotherapy for Ewing sarcoma

**DOI:** 10.1111/bjd.12574

**Published:** 2013-12-02

**Authors:** E Higgins, M Capra, ME Schwartz, FJD Smith, WHI McLean, AD Irvine

**Affiliations:** 1 Department of Paediatric Dermatology, Our Lady's Children's HospitalCrumlin, Dublin, Ireland; 2 Department of Oncology, Our Lady's Children's HospitalCrumlin, Dublin, Ireland; 3 Pachyonychia Congenita ProjectSalt Lake City, UT, U.S.A; 4 Centre for Dermatology and Genetic Medicine, College of Life Sciences and College of Medicine, Dentistry and Nursing, University of DundeeDundee, DD1 5EH, U.K; 5 National Children's Research Centre, Our Lady's Children's HospitalCrumlin, Dublin, Ireland; 6 Department of Clinical Medicine, Trinity College DublinDublin, Ireland

Dear Editor, A 15-year-old girl with pachyonychia congenita (PC) confirmed by genetic testing presented with pain and a localized subcutaneous swelling on her right back. Biopsy confirmed Ewing sarcoma of the posterior 10th rib. Preoperative tumour-reduction chemotherapy was commenced as per the Euro EWING 99 protocol, with vincristine, ifosfamide, doxorubicin and etoposide.^1^ Ifosfamide was subsequently replaced by cyclophosphamide, as per protocol, due to potential renal tubule toxicity (Table 1).^1^ Surgical resection was performed followed by radiotherapy to the primary site.

**Table 1:** Chemotherapy regimen

Week of regimen	Euro EWING 99 Protocol	
Week 12 (Figs 1b and 2, after cycle 4)	Cycles 1–4	Vincristine, ifosfamide, doxorubicin, etoposide (VIDE) × 4
Week 16 (Fig. 1c, after cycle 6)	Cycles 5–6	Vincristine, cyclophosphamide, doxorubicin, etoposide (VCDE) × 2. Ifosfamide replaced by cyclophosphamide due to potential tubulopathy
	Surgical resection performed after cycle 6	
	Cycles 7–15: maintenance cycles. Radiotherapy performed after cycle 8	Vincristine, actinomycin, cyclophosphamide (VAC) × 8
	Cumulative doses	Vincristine, ifosfamide 36 g m^−2^, doxorubicin 360 mg m^−2^, etoposide 2340 mg m^−2^, cyclophosphamide 11·625 g m^−2^
Week 92 (Fig. 1d), 36 weeks after completion of chemotherapy		

Prior to chemotherapy, our patient had painful focal plantar keratoderma, in a distribution characteristic of PC, affecting the weight-bearing areas of both soles. Each area had a rim of surrounding erythema. Mild toenail dystrophy was present (Fig. 1a). The painful keratoderma had a significant impact on the girl's quality of life, with pain and impaired mobility. She had a known keratin 16 mutation: *KRT16* c.374A>G; K16 p.Asn125Ser. Previous treatment with keratolytic agents, including urea, salicylic acid and tar-based preparations, along with regular intense podiatric therapy, had resulted in very limited improvement. Six-monthly plantar botulinum toxin injections helped with pain and were ongoing prior to her illness.

**Fig 1:** Clinical features of the focal keratoderma of pachyonychia congenita, (a) prior to treatment, (b) after 12 weeks, (c) after 16 weeks and (d) after 92 weeks (36 weeks after chemotherapy completion).

A dramatic improvement in the keratoderma was noted following chemotherapy induction. Six weeks into chemotherapy, shedding of large areas of hyperkeratotic plaques commenced. A marked improvement was noted at 12 weeks (Figs 1b and 2), and this improvement continued at 16 weeks (Fig. 1c). There was virtually no residual keratoderma during maintenance chemotherapy and on completion of treatment. The clearance was maintained for a further 9 months following remission of the Ewing sarcoma and cessation of all chemotherapy (Fig. 1d), with minimal areas of keratoderma redeveloping at pressure sites after this.

**Fig 2:** Close-up detail of hyperkeratotic skin shedding after 12 weeks of chemotherapy.

The precise mechanism of this observed effect is not clear, and as far as we know this clinical observation has not been previously noted. The mechanisms may have some analogies to the better understood chemotherapy-induced alopecia (CIA). CIA is a frequent toxic side-effect of cytotoxic cancer therapy. It results as a direct toxic effect on the rapidly dividing cells of the hair follicle. A major characteristic of the anagen hair follicle is that the epithelial compartment undergoes proliferation, with the greatest proliferation activity at the bulb matrix cells building up the hair shaft. The abrupt cessation of mitotic activity leads to the weakening of the partially keratinized proximal portion of the hair shaft, with narrowing and subsequent breakage within the hair canal.^2^ As a result, hair shedding begins at 1–3 weeks from anagen follicles (anagen effluvium), and is complete at 1–2 months after chemotherapy initiation. The frequency and severity of CIA differs among chemotherapeutic drug classes, with the commonest agents including topoisomerase agents such as doxorubicin and daunorubicin, antimicrotubule agents such as paclitaxel, and alkylators such as cyclophosphamide and ifosfamide.^3^ CIA with permanent alopecia has been reported, particularly with taxanes.^4^

The epidermis of ridged or palmoplantar skin expresses a complex pattern of keratins due to the greater stress it must withstand. Specific expression patterns of keratins K6, K16 and K17 have been demonstrated in ridged skin, suggestive of regional adaptation patterns resulting in high proliferative activity of normal ridged skin.^5^ Mutations in suprabasal keratins lead to hyperkeratosis of specific epithelia. Mutations in the site-specific keratins K6a, K6b, K6c, K16 or K17 lead to phenotypic variants of PC, with painful hyperkeratosis of the soles and other sites where these keratins are expressed, such as the nails and oral mucosa.^6^ Thus, chemotherapy may improve hyperkeratosis primarily by an antiproliferative mechanism. The disease mechanisms in PC are thought to initiate with cell lysis (blistering, when lysed cells coalesce) due to dominant negative mutations in the above keratins impairing the structural mechanics of the cytoskeleton of the suprabasal cells in the relevant skin sites. This observed effect suggests that a hyperproliferative response may have a greater role in pachyonychia than previously recognized. The same hyperproliferative mechanism may also be important in other dominant negative keratin disorders such as keratinopathic ichthyosis. We recognize that other mechanisms could possibly explain this observed effect, for example bed rest may reduce local stress and friction to hyperkeratotic areas; however, anecdotal data collected through the International Pachyonychia Congenita Registry (IPCRR) suggest that bed rest results in little improvement in most cases. Chemotherapy could also inhibit inflammatory cytokines implicated in pain pathogenesis and possibly hyperkeratosis.

While our patient experienced a dramatic resolution of her symptoms during chemotherapy, we are aware of other patients with PC on different chemotherapy regimens (for breast cancer) who have not experienced this dramatic benefit (IPCRR, unpublished data), so the response may be regimen specific. Clearly such a regimen is too toxic to be applied to patients with PC in the broader sense, but our hope is that this observation may stimulate thinking as to how to harness this positive effect in a safe and localized fashion.

## References

[1] Juergens C, Weston C, Lewis I (2006). Safety assessment of intensive induction with vincristine, ifosfamide, doxorubicin, and etoposide (VIDE) in the treatment of Ewing tumours in the EURO-E.W.I.N.G. 99 clinical trial. Pediatr Blood Cancer.

[2] Trueb RM (2009). Chemotherapy-induced alopecia. Semin Cutan Med Surg.

[3] Chon SY, Champion RW, Geddes ER, Rashid RM (2012). Chemotherapy-induced alopecia. J Am Acad Dermatol.

[4] Palamaras I, Misciali C, Vincenzi C (2011). Permanent chemotherapy-induced alopecia: a review. J Am Acad Dermatol.

[5] Swensson O, Langbein L, McMillan JR (1998). Specialized keratin expression pattern in human ridged skin as an adaptation to high physical stress. Br J Dermatol.

[6] McLean WH, Hansen CD, Eliason MJ, Smith FJ (2011). The phenotypic and molecular genetic features of pachyonychia congenita. J Invest Dermatol.
